# Hypocretin in median raphe nucleus modulates footshock stimuli-induced REM sleep alteration

**DOI:** 10.1038/s41598-019-44731-0

**Published:** 2019-06-03

**Authors:** Yi-Tse Hsiao, Yun Lo, Pei-Lu Yi, Fang-Chia Chang

**Affiliations:** 1 Department of Veterinary Medicine, School of Veterinary Medicine, National Taiwan University, Taipei, Taiwan; 2 Department of Sport Management, College of Tourism, Leisure and Sports, Aletheia University, Taipei, Taiwan; 3 Graduate Institute of Brain & Mind Sciences, College of Medicine, National Taiwan University, Taipei, Taiwan; 4 Graduate Institute of Acupuncture Science, College of Chinese Medicine, China Medical University, Taichung, Taiwan

**Keywords:** Hypocretin, REM sleep

## Abstract

Stress is one of major factors that cause sleep problems. Hypocretin represents a stress-related neuropeptide and is well known in maintaining physiological wakefulness. The hypocretinergic neurons originate in the lateral hypothalamic area (LHA) and transmit to several brain regions, including the median raphe nuclei (MRNs). The MRNs modulate both fear responses and sleep-wake activity; however, it remains unclear whether stress alters the levels of hypocretin to regulate MRNs and consequently disrupt sleep. In this paper, we employed the inescapable footshock stimuli (IFS) as a stressor and hypothesized that the IFS-induced sleep disruption is mediated by increased hypocretins in the MRNs. Our results demonstrate that the concentrations of hypocretin in the hypothalamus increased after IFS. Rapid eye movement (REM) sleep was reduced after footshock, and microinjection of non-selective hypocretin receptor antagonist TCS-1102 into the MRNs blocked the IFS-induced decrease of REM sleep. Furthermore, administration of hypocretins into the MRNs mimicked the IFS-induced REM sleep reduction. These results conclude that the increased levels of hypocretins in the MRNs mediate the IFS-induced REM sleep reduction.

## Introduction

Both brain functions and mental processes are influenced by stress^1,2^. Stress experiences in the daytime may cause sleep disturbances during the night^3^. Animal studies have demonstrated that acute and chronic stressors alter sleep-wake activities. Different kinds of stressors cause sleep disturbances differently^4–7^. For instance, the inescapable footshock stimuli (IFS) significantly reduces rapid eye movement (REM) sleep, while the escapable footshock stimuli increases REM sleep^7,8^. Our previous study has also elucidated that the amount of REM sleep is decreased by repeatedly and consecutively applying the combination of two different stressors, the open field and the elevated plus-maze^9^. REM sleep seems to be more susceptible to influence under stress.

Certain neuropeotides, such as corticotropin-releasing hormone (CRH) and hypocretins (hcrts)^10,11^, modulate the physiological sleep-wake activities in addition to the stress responses. There are two types of hcrts, the hcrt-1 and hcrt-2. Our pervious result has shown that administration of hcrts into the bilateral median raphe nuclei (MRNs) enhances the theta power of electroencephalograms (EEGs), an index of anxiety level, which is similar to the result of rats which received the IFS^12^. Furthermore, the hcrt receptor antagonist blocks IFS-induced augmentation of theta waves^12^. These results demonstrate that hcrts in the MRNs mediate IFS-induced anxiety and stress response^12^. It’s well known that hcrts play an important role in maintaining animal’s physiological wakefulness^13,14^. An insufficiency of hcrts causes a symptom of sudden sleep with an increased REM sleep^15,16^. Several brain regions that regulate stress responses or sleep-wake activity are abundant of hcrts and hcrt receptors. These brain structures include the paraventicular nucleus (PVN)^17,18^, the laterodorsal tegmental nucleus (LDT)^18,19^, the pedunculopontine tegmental nucleus (PPT)^18,19^, the hypothalamic preoptic nuclei^17,18^, the raphe nuclei^17–19^, and the MRNs^20^. Although we previously demonstrated hcrts mediate IFS-induced stress response, the role of hcrts in IFS-induced sleep alteration has never been determined.

The MRNs contain the bulk of serotonergic neurons, which project to the hypothalamus and limbic system structures^21^ and modulate stress, fear, and anxiety responses^21–25^. The MRNs also mediate the fundamental sleep regulation^26–29^. The firing rate of serotoninergic neurons is alleviated during non-REM (NREM) sleep and increased during REM sleep^30^. The activity of GABAergic interneruons in the MRNs is higher during the period after REM sleep deprivation^31^. Furthermore, the MRNs are believed to convey non-photic stimuli to the suprachiasmatic nuclei (SCN) of the hypothalamus and regulate circadian rhythm^28,29^. A study of *in situ* hybridization has also demonstrated that the expression of hcrt receptor mRNA is abundant in the MRNs^18^. The hypocretinergic neurons in the LHA of hypothalamus project a large number of axons to the MRN^17^. Our previous findings have demonstrated hcrts mediate IFS-induced stress response. Based on the rationales that hcrts mediate stress response^12^, the MRN receives hypocretinergic afferents from LHA^17^, and the MRN regulates REM sleep activity^30^, we herein hypothesized that hcrts in the MRNs play an important role in the IFS-induced REM sleep disruption. We performed IFS to induce acute stress in rats and determined the consequent sleep disturbances. We proposed that stress, especially for the IFS stressor, increases the releases of hcrts in the MRNs and subsequently mediates the IFS-induced sleep alterations. To test this hypothesis, the expressions of hcrts and prepro-hcrt in the LHA after IFS were determined, the concentrations of hcrts in the hypothalamus after IFS were quantified by enzyme-linked immunosorbent assay (ELISA), hcrt receptor agonists or antagonist were administered into the bilateral MRNs with the IFS, and the sleep-wake activities were measured. In this paper, we demonstrated the neuronal mechanism of sleep disturbance underlying the IFS stress.

## Results

### IFS altered sleep-wake activity

**Figure 1:**
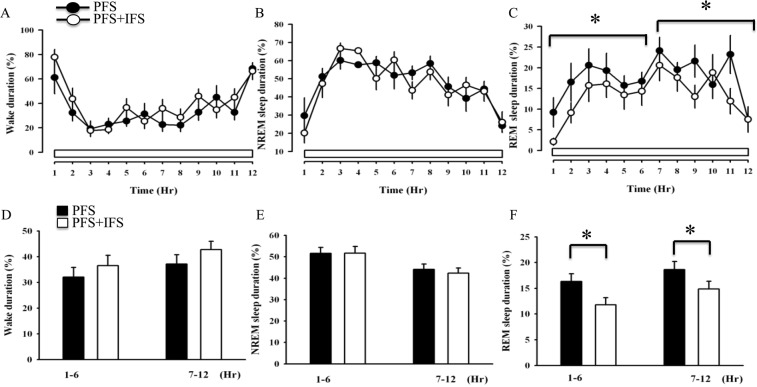
The effects of IFS on sleep alterations. Closed circles and black bars represent the values obtained after administration of PFS, and open circles and white bars depict the values acquired after administration of PFS + IFS. *p < 0.05 PFS + IFS vs. PFS.

In order to compare the effects with those which received hypocretin (dissolved in pyrogen-free saline; PFS) and those who received hypocretin receptor antagonist (dissolved in 0.5% saline and 20% D-α-tocopherol polyethylene glycol 1000 succinate; TPGS), rats were microinjected PFS or TPGS into the MRN before the IFS. The time spent in NREM sleep and waking showed no significant change after the IFS when compared to the data obtained after PFS (Fig. 1A,B,D,E). However, the percentage of REM sleep during the 12-hr light period decreased from 17.5 ± 1.1% to 13.3 ± 1.03% (F_(1,83)_ = 10.77, *p* < 0.001; Fig. 1C,F) after the IFS. Analysis of first 6 hours of REM sleep revealed that the percentage of REM sleep significantly reduced from 16.6 ± 1.5% to 11.8 ± 1.5% (F_(1,41)_ = 5.39, *p* < 0.05, Fig. 1F). The percentage of REM sleep during the last 6 hours of the light period was reduced from 18.64 ± 1.5% to 14.8 ± 1.5% (F_(1,41)_ = 5.36, *p* < 0.05, Fig. 1F). The reason to split the data into separate 6-hr bins (the first 6 hours and the last 6 hours) is because the manipulation to alter sleep-wake activity may only last for few hours, rather than  12 hours. Therefore, we always performed the statistical analysis across the  12 hours first, and then further analyzed data from the two separate 6-hr time slots. This is common in performing statistical analysis in sleep research^10^.

**Figure 2:**
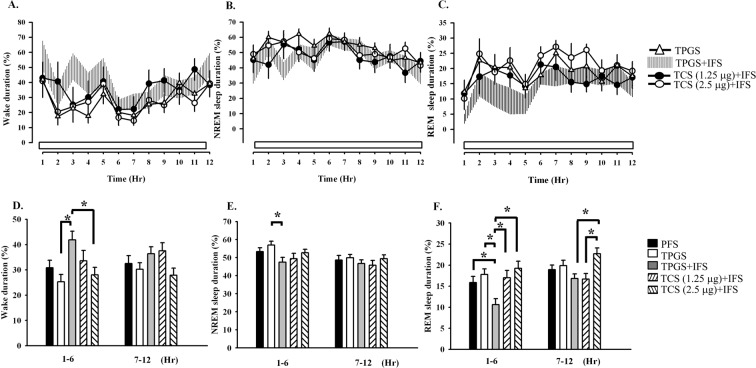
The effects of IFS on sleep alterations and hcrt receptor antagonist (TCS-1102) on IFS-induced sleep alterations. From A to C, the symbols of open triangle, shaded areas, closed circle and open circle are respectively representing the values obtained after TPGS, TPGS + IFS, TCS-1102 (1.25 μg) + IFS, and TCS-1102 (2.5 μg) + IFS. In panels D, E and F, the bars of histograms from left to right represent the PFS control, TPGS control, TPGS + IFS, TCS-1102 (1.25 μg) + IFS, and TCS-1102 (2.5 μg) + IFS. *p < 0.05.

Neither NREM sleep, REM sleep nor wakefulness was altered by administration of 20% TPGS when comparing to the effect after giving PFS (Fig. 2D–F), suggesting that 20% TPGS does not alter sleep-wake activity. The time spent in NREM sleep was significantly decreased during the first 6 hours after the IFS (Fig. 2B,E). The percentage of NREM sleep during hours 1–6 was decreased from 56.9 ± 2.2% obtained from 20% TPGS to 47.5 ± 2.6% after 20% TPGS + IFS (F_(1,41)_ = 4.285, p < 0.01; Fig. 2E). The percentage of REM sleep during the first 6-hr light period decreased from 17.8 ± 1.3% obtained after 20% TPGS to 10.6 ± 1.4% (F_(1, 41)_ = 9.383, *p* < 0.01; Fig. 2C,F) after the IFS. Wakefulness was significantly increased during the first 6-hr of the light period after the IFS (Fig. 2A,D). The percentage of wakefulness during hours 1–6 was increased from 25.3 ± 2.9% obtained from 20% TPGS to 41.8 ± 3.5% after 20% TPGS + IFS (F_(1,41)_ = 7.048, p < 0.01; Fig. 2D). Sleep-wake activities, including wakefulness, NREM sleep and REM sleep were not altered during hours 7–12 of the light period after the IFS (Fig. 2).

Analysis of sleep-architecture parameters across the 6-h light period revealed that the IFS-induced reduction of REM sleep was primarily due to reduction in the number of REM sleep bouts. The number of REM sleep bouts decreased from 5.3 ± 0.5 obtained after 20% TPGS to 2.9 ± 0.6 (F_(1,6)_ = 10.84, *p* < 0.05; Table 1) after the 20% TPGS + IFS. IFS prolonged the latencies of entering NREM sleep and REM sleep, although they did not reach statistical significance (Table 1). Slow wave activities (SWAs) during NREM sleep also were not altered by the IFS; the SWAs during NREM sleep in the 12-h light period after PFS administration and IFS manipulation were 1165.6 ± 12.4 μV^2^/Hz and 1165.1 ± 10.9 μV^2^/Hz, respectively.

**Table 1:** Effects of IFS, TPGS, TCS-1102 + IFS, hcrt-1 and hcrt-2 on the sleep-wake architecture parameters in rats.

Manipulation^e^	Hour	L:D cycle^f^	Number of Bouts^a^			Bout Duration^b^			Transitions^c^	Latency^d^	
			Wake^g^	NREMS^g^	REMS^g^	Wake	NREMS	REMS		NREMS	REMS
PFS	1–12	L	5.6 ± 0.5	10.8 ± 0.7	5.0 ± 0.3	5.1 ± 1.1	2.6 ± 0.1	2.0 ± 0.2	40.3 ± 3.8	34.0 ± 10.7	56.8 ± 18.0
PFS + IFS	1–12	L	5.9 ± 0.8	10.4 ± 0.9	3.8 ± 0.3*	5.8 ± 1.0	2.8 ± 0.2	1.9 ± 0.3	37.4 ± 2.9	44.8 ± 12.7	72.0 ± 13.1
PFS	1–6	L	5.0 ± 0.6	9.9 ± 0.9	4.6 ± 0.5	3.8 ± 0.5	3.4 ± 0.3	1.9 ± 0.2	26.2 ± 2.3	6.9 ± 1.8	31.7 ± 9.4
20% TPGS	1–6	L	3.6 ± 0.3	9.8 ± 0.8	5.3 ± 0.5	4.5 ± 0.7	3.5 ± 0.3	1.9 ± 0.1	23.3 ± 1.7	13.5 ± 4.7	34.7 ± 9.3
20% TPGS + IFS	1–6	L	7.5 ± 0.6*^,#^	10.8 ± 1.1	2.9 ± 0.6^#^	3.6 + 0.7	2.6 ± 0.3	1.7 ± 0.2	26.5 ± 3.3	17.6 ± 3.4*	63.4 ± 16.1
1.25 μg TCS + IFS	1–6	L	4.6 ± 0.8	9.7 ± 0.6	5.1 ± 0.9^§^	4.7 ± 1.1	3.1 ± 0.3	1.8 ± 0.3	23.5 ± 2.1	8.8 ± 3.4	36.5 ± 12.4
2.5 μg TCS + IFS	1–6	L	3.6 ± 0.6§	9.6 ± 0.5	5.4 ± 0.8^§^	5.2 ± 0.7	3.3 ± 0.1	2.0 ± 0.1	23.1 ± 1.8	7.6 ± 2.1^§^	36.5 ± 12.8
PFS	1–6	L	6.4 ± 0.5	11.0 ± 0.8	5.0 ± 0.5	4.7 ± 1.8	2.9 ± 0.1	1.4 ± 0.1	37.2 ± 3.0	21.8 ± 10.5	49.1 ± 18.6
1 μg hcrt-1	1–6	L	5.3 ± 0.5	10.9 ± 1.2	3.9 ± 0.5	10.1 ± 1.1*	2.7 ± 0.3	1.0 ± 0.1	39.9 ± 4.2	75.2 ± 7.0*	130.5 ± 12.3*
10 μg hcrt-1	1–6	L	5.5 ± 0.6	9.4 ± 0.7	2.6 ± 0.7	16.4 ± 2.1*^,☆^	2.2 ± 0.2	0.7 ± 0.1*	32.3 ± 2.6	96.1 ± 13.0*	164.9 ± 26.1*
1 μg hcrt-2	1–6	L	5.1 ± 0.6	10.2 ± 0.9	5.0 ± 0.9	5.7 ± 1.1	3.2 ± 0.4	1.1 ± 0.1	35.6 ± 2.1	36.9 ± 12.8	70.0 ± 14.9
10 μg hcrt-2	1–6	L	6.4 ± 0.6	10.5 ± 0.7	3.5 ± 0.5	7.0 ± 1.5	2.7 ± 0.2	0.9 ± 0.2*	35.6 ± 2.4	46.0 ± 11.2	101.2 ± 23.1

Values are Means ± S.E.M. *p < 0.05 vs. PFS; ^#^*p* < 0.05 vs. TPGS. ^§^*p* < 0.05 vs. TPGS + IFS. ^☆^*p* < 0.05 vs. 1 μg hcrt-1.

^a^Number of bouts per hour (mean ± SEM) for each vigilance state.

^b^Mean ± SEM bout duration (min) for each vigilance state.

^c^Number of transitions from one behavioral state to another (mean ± SEM) per hour.

^d^Mean ± SEM sleep latency (min) for each vigilance state.

^e^Experimental manipulation.

^f^Period of the light:dark cycle immediately prior to which injections were given: L = light period.

^g^Vigilance states: WAKE, wakefulness; NREMS, non-rapid eye movement sleep; REMS, rapid eye movement sleep.

### Hypocretin receptor antagonist TCS-1102 blocked IFS-induced reduction of REM sleep

To determine the involvement of hypocretin in IFS-induced decrease of REM sleep, we microinjected a hypocretin receptor antagonist, TCS-1102, directly into the MRNs. We found that TCS-1102 dose-dependently blocked the increase of wakefulness and the decrease of REM sleep induced by the IFS (Fig. 2D,E). High dose (2.5 μg) of TCS-1102 significantly reduced the IFS-induced increase of wakefulness to 28.0 ± 3.0% during the first 6 hours of the light period (F_(1,41)_ = 7.048, p < 0.01 vs. TPGS + IFS; Fig. 2D). Low dose (1.25 μg) and high dose (2.5 μg) of TCS-1102 blocked the IFS-induced decrease of REM sleep during the first 6 hours. The percentages of time spent in REM sleep during the first 6-h after administrations of 1.25 μg and 2.5 μg TCS-11102 were 17.0 ± 1.7% (p < 0.01 vs. TPGS + IFS) and 19.2 ± 1.7% (p < 0.01 vs. TPGS + IFS; Fig. 2F), respectively. High dose (2.5 μg) of TCS-1102 also increased REM sleep during the second 6 hours when compared to that received TPGS + IFS (Fig. 2F). TCS-1102 did not have effect on IFS-induced decrease of NREM sleep (Fig. 2E). Analysis of sleep-architecture parameters across the 6-h light period revealed that TCS-1102 blocked IFS-induced REM sleep suppression was primarily due to the increases in the number of REM sleep bouts (Table 1).

We also need to verify whether or not the microinjected hypocretin receptor antagonist *per se* alters REM sleep. We then microinjected 1.25 μg or 2.5 μg TCS-1102 into the MRNs prior to the beginning of the light period and revealed no significant influence on the sleep-wake activity during the light period when comparing with that obtained after administration of vehicle solution. The total amount of time spent in NREM sleep during the light period after receiving vehicle solution (TPGS), 1.25 μg TCS-1102 and 2.5 μg TCS-1102 was 48.5 ± 2.3%, 46.6 ± 1.8% and 46.7 ± 1.6%, respectively. The total amount of time spent in REM sleep after receiving vehicle solution (TPGS), 1.25 μg TCS-1102 and 2.5 μg TCS-1102 was 17.8 ± 1.4%, 17.0 ± 0.9% and 15.5 ± 0.8%, respectively.

### Microinjection of hcrt-1 or hcrt-2 into the MRNs reduced REM sleep

**Figure 3:**
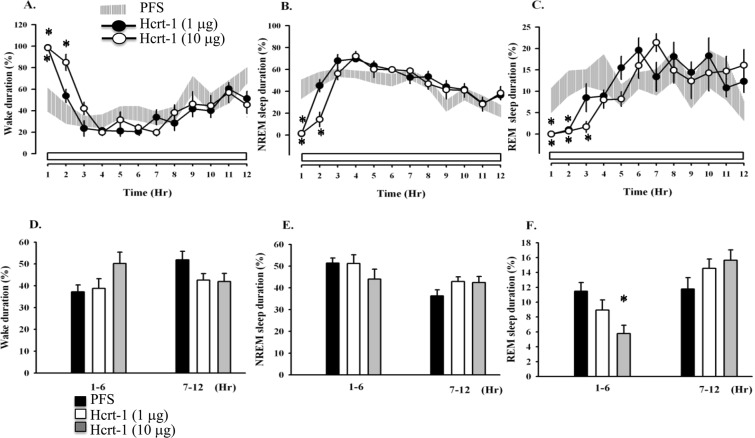
The effects of hcrt-1 on sleep. Shaded areas represent the values obtained from the PFS control, closed circles depict values acquired after microinjection of hcrt-1 (1 μg), and open circles demonstrate those gained after microinjection of hcrt-1 (10 μg). The bars of histograms from left to right represent the values obtained from the PFS control, low dose hcrt-1 (1 μg), and high dose hcrt-1 (10 μg). *p < 0.05 vs. PFS injection.

**Figure 4:**
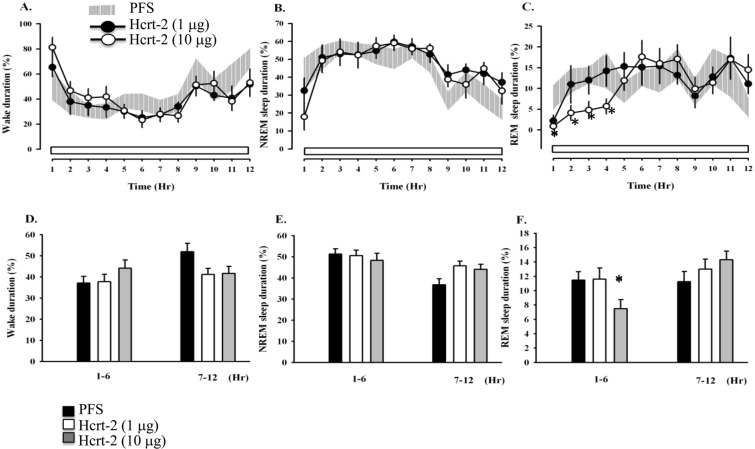
The effects of hcrt-2 on sleep. Shaded areas represent the values obtained from the PFS control, closed circles depict values acquired after microinjection of hcrt-2 (1 μg), and open circles demonstrate those gained after microinjection of hcrt-2 (10 μg). The bars of histograms from left to right represent the values obtained from the PFS control, low dose hcrt-2 (1 μg), and high dose hcrt-2 (10 μg). *p < 0.05 vs. PFS injection.

If the REM sleep suppression after IFS was mediated by the increase of hcrt levels in the MRN, administration of hcrts directly into the MRN should produce similar REM sleep suppression. Since hypocretin was dissolved in PFS, we compared the data with the PFS control. We microinjected hcrt-1 (1.0 and 10 μg) into the MRNs 15 minutes prior to the beginning of light period and found that 10 μg hcrt-1 significantly reduced the percentage of REM sleep during hours 1–6 (F_(2,82)_ = 7.11, *p* < 0.01 vs. PFS injection; Fig. 3C,F), but did not affect REM sleep during hours 7–12 of the light period. That’s because the REM sleep suppression induced by microinjection of hcrt-1 directly into the MRN could only last few hours and the effect disappeared after hcrt-1 had been washed out. The Scheffe *post hoc* analysis comparison indicated that 10 μg of hcrt-1 significantly reduced REM sleep from 11.5 ± 1.2% obtained after PFS administration to 5.8 ± 1.1% (*p* < 0.01, the grey bar vs. the closed bar; Fig. 3C,F). In addition to the reduction of REM sleep, NREM sleep was reduced and wakefulness was increased during hours 1–2 after the administration of hcrt-1 (Fig. 3A,B). Administration of hcrt-2 exhibited a similar effect of REM sleep reduction as that obtained after hcrt-1 administration. Hcrt-2 (10 μg) decreased REM sleep to 7.5 ± 1.3% (*p* < 0.05, the grey bar vs. the closed bar; Fig. 4C,F) during hours 1–6.

### Hcrts altered sleep architecture

Further analyzing the alterations in sleep architectures, we found that the effects of hcrt-1 and hcrt-2 occurred mainly during the first 6 hours after the light period. Microinjections of hcrt-1 significantly reduced the average bout number of REM sleep (F_(2,12)_ = 5.15, *p* < 0.05; Table 1); in contrast, hcrt-2 exhibited no significant alteration. The Scheffe *post hoc* analysis comparison also indicated that the bout duration of REM sleep was significantly changed by 10 μg hcrt-1 (*p* < 0.05) and 10 μg hcrt-2 (*p* < 0.05). The latency of NREM sleep (F_(2,12)_ = 13.38, *p* < 0.001) and REM sleep (F_(2,12)_ = 14.59, *p* < 0.01) was dose-dependently prolonged after microinjection of hcrt-1 (Table 1).

### IFS increased prepro-hypocretin (prepro-hcrt), hcrt-1 and c-fos immunoreactive (IR) neurons in LHA

**Figure 5:**
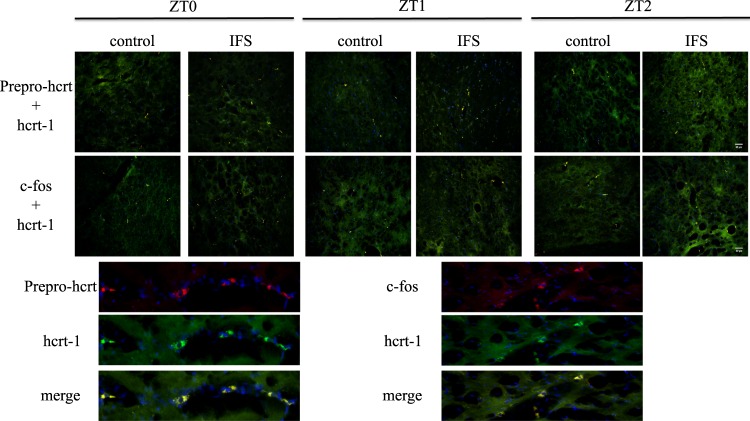
The expression of prepro-hcrt, hcrt-1 and c-fos IR neurons. The expression of prepro-hcrt and c-fos was indicated by the red fluorescence, the hcrt-1 was delineated by the green fluorescence, the merge image of prepro-hcrt and hcrt-1 was shown in yellow fluorescence, and the merge image of c-fos and hcrt-1 was also shown in yellow fluorescence. The cell nuclei were indicated as the DAPI blue fluorescence.

If hcrt mediated IFS-induced REM sleep alteration, the expression of hcrts in LHA will be increased after the IFS. Our result demonstrated that the most expression of prepro-hcrt (red fluorescence) is co-localized (yellow fluorescence) with the hcrt-1 (green fluorescence) in LHA hypocretinergic neurons. In a series of brain slices from the rostral to caudal LHA region, we found the expressions of prepro-hcrt and hcrt-1 were simultaneously enhanced at the zeitgeber time (ZT) 0 and ZT2 after rats received IFS. The cell number, which exhibited the merging (yellow) fluorescence of prepro-hcrt and hcrt-1, was increased from 12,003 to 13,640, from 11,049 to 11,932, from 12,929 to 16,323 at the ZT0, ZT1 and ZT2 (Fig. 5), respectively.

Furthermore, the IFS increased c-fos expression in the LHA; however, there are more c-fos expressing neurons beyond the hcrt-1 expressing neurons (Fig. 5). This result suggests that the IFS increased neuronal activation not only in the hypocretinergic neurons in the LHA, but also other cell type(s) of neuron(s).

### Hypocretin concentrations in hypothalamus was quantified by ELISA

We further quantified the concentrations of hypocretin in the hypothalamus in rats with or without the IFS. Our results indicated that the concentrations of hypocretin were significantly increased in ZT0 and ZT2 after the IFS when compared with those obtained with no IFS. The concentrations of hypocretin increased from 74.64 ± 18.54 pg/ml obtained after control to 147.25 ± 40.89 pg/ml (unpaired t-test, p < 0.05, n = 6) after the IFS at ZT0, and it increased from 81.30 ± 23.63 pg/ml obtained after control to 167.69 ± 7.37 pg/ml (unpaired t-test, p < 0.05, n = 6) after the IFS at ZT2 (Table 2).

**Table 2:** The concentrations of hypocretin in the hypothalamus at ZT0, ZT1 and ZT2 obtained from control and IFS.

	ZT0	ZT1	ZT2
CONTROL	76.46 ± 18.54	113.84 ± 32.34	81.30 ± 23.63
IFS	147.25 ± 40.89*	187.52 ± 13.58	167.69 ± 7.37*

Values are Means ± S.E.M. *p < 0.05 vs. control.

### Microinjection did not cause lesion in MRNs

**Figure 6:**
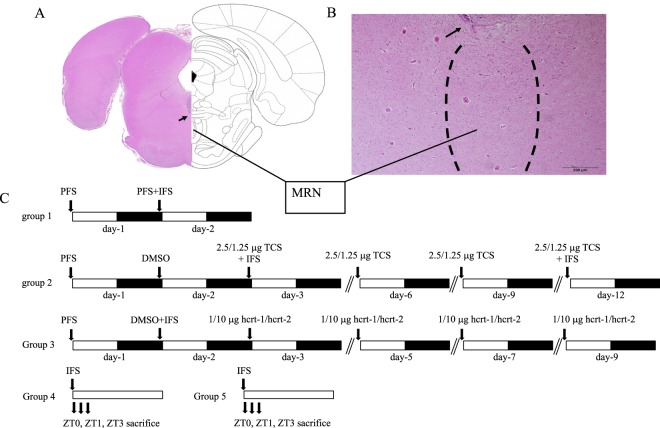
(**A**) Left side: the histological slide of the MRNs. Right side: rat brain coordinates. (**B**) 200X magnification of the MRNs. Arrow depicts the end of the cannula track. (**C**) The diagram of the experimental protocol. Close bar indicates the dark period and the open bar represents the light period of the 12:12 hr light:dark cycle. Arrow depicts the timing of microinjection, IFS represents the inescapable footshock stimulation.

Histological examination indicated only a minor lesion was observed on the top margin of the MRN, as shown in Fig. 6A,B, after rats received multiple 0.5 μl microinjections. Arrows in Fig. 6A,B indicate the lesion. The MRNs remained intact after receiving multiple microinjections.

## Discussion

The causes of anxiety disorders in human beings are complex and patients’ sleep patterns may be influenced by medications or disease comorbidity. Establishing a suitable animal model is an essential solution to study anxiety disorders. Applying different stressors at the light period to rats or mice usually results in decreases of REM sleep^6^. Employing physical restraint^32^, open field^9,33^, IFS^7^, and contextual fear reduced REM sleep with a minimal effect on NREM sleep^34^. Studies demonstrate that REM sleep in patients with anxiety disorders (e.g., panic disorder) and stress-related disorders, such as post-traumatic stress disorder (PTSD), is disrupted. Decreases of REM sleep latency has been noted in patients with panic disorder^35^. Patients suffering from PTSD show a symptom of REM sleep interruption and a high correlation of nightmares with REM sleep disturbance^36^. The variety of changes in REM sleep has been reported, including enhancement, the same or reduction, in the PTSD patients^37–40^. Our present data showed that the IFS prior to the beginning of the light period reduced the percentage of REM sleep during the light period. The circadian time point when the stressor is applied to animals is critical. For example, employing physical restraint at the beginning of light period decreases REM sleep^32^; in contrast, immobilization during the dark period increases the subsequent REM sleep in rats^41,42^. These reports suggest that the mechanisms of stress-induced sleep alternations are complicated. Moreover, stressors alter the fluctuations of neurotransmitters or neuropeptides, which control sleep via the homeostatic process. For example, the CRH secretion is elevated by the IFS and consequently decreases REM sleep^8^. Other neurotransmitters or neuropeptides may also involve in stressor-related REM sleep alteration, but need to be further investigated. In this paper we focused on the influence of hcrts.

The projection of hypocretinergic neurons and the distribution of hcrt receptors imply that hcrts may modulate the stage of vigilance, especially REM sleep. The ascending reticular activating system in the brain stem expresses high density of hcrt receptors^18,19^. The hypocretinergic neurons project widely to the brain areas to regulate wakefulness and REM sleep. These brain structures include the REM–off neurons (e.g., locus coeruleus, the dorsal raphe nucleus (DRN)) and the REM-on nuclei, such as the PPT and LDT^17–19^. Administration of hcrt-1 into the LDT or locus coeruleus decrease the number of REM episodes^13,14^. Our result has indicated that IFS reduced the percentage of REM sleep by interfering with the number of bouts. Therefore, we designed this experiment to examine the involvement of hcrts in stress-induced sleep disturbance. Since the time of stressor occurrence is critical, we performed IFS and administered TCS-1102 prior to the beginning of light period when the fluctuation of hcrt level is at the lowest in LHA^43^. Our data demonstrated that the expression of prepro-hcrt was co-localized with the hcrt-1 in the LHA, and both the prepro-hcrt IR and hcrt-1 IR were simultaneously increased at ZT0 and ZT2 after rats received the IFS. The IFS also increased c-fos expression in the LHA; however, there are more c-fos expressing neurons beyond the hcrt-1 expressing neurons. This result suggests that the IFS increased neuronal activation not only in the hypocretinergic neurons in the LHA, but also other cell type(s) of neuron(s). Some researches are consistent with our current result. For example, cold stress and immobilization enhance the c-fos signals of hcrt-1 neurons and prepro-hcrt mRNA in LHA^44,45^.

The MRN neurons express moderate density of hcrt receptor 1 and hcrt receptor 2^18,46^, and are innervated by the hypocretinergic neurons from LHA^47^. MRN is believed to modulate fear, anxiety responses^21–25^ and sleep-wake activities^26–29^. We attempted to utilize hcrt receptor antagonist TCS-1120 to clarify the involvement of MRN hcrts in the stress-induced REM sleep disruption. However, the limitation of our experiment is that TCS-1120 can hardly be dissolved in water. In our current study, we dissolved TCS-1102 in a 20% TPGS solution. We first determined whether 20% TPGS alters sleep-wake activity, and found that TPGS exhibited no effect on sleep-wake activity. Our result further indicated that TCS-1102 dose-dependently blocked the IFS-induced decrease of REM sleep, suggesting that hypocretins mediate the effect.

Our results demonstrate that hcrt-1 possesses stronger REM sleep suppression than hcrt-2 does when injecting hcrt1 and hcrt2 into the MRNs. The MRNs have been reported to contain high densities of hcrt-IR fibers and similar quantities of hcrt receptor type-1 and type 2 expression in rats^17,18,46^. A possible explanation may be due to the fact that hcrt-1 acts on both hcrt receptor type 1 and type 2, whereas hcrt-2 selectively binds to the hcrt receptor type 2. Nevertheless, our results indicate that hcrt-1 affects sleep activities more than hcrt-2 does. Administration of hcrts into the MRNs did not influence REM sleep during hours 7 to 12 when compared with the effect of IFS. The differences may be because the effect after administering a bolus (0.5 μl) of hcrt into the MRNs is washed out soon, rather than the long lasting effect influenced by the IFS stressor.

The activity of hypocretinergic neurons acts as an important regulator in the vigilance. During REM sleep, the activities of hypocretinergic neurons is at its lowest^47^. The hypocretinergic neurons are partially activated and stimulate REM-off nuclei during NREM sleep, and the activity is at its highest during wakefulness^47^. Our results indicate that IFS prior to the light period interferes the homeostasis of hcrts in the MRNs and suppresses REM sleep.

In summary, hcrts in the MRNs not only modulate the generation of theta waves, an index of anxiety, as we published previously^12^, but also regulate REM sleep in the stress condition. Our results demonstrate that maintaining the homeostasis of hcrts in the MRNs may improve the stress-induced sleep disturbance, especially in REM sleep.

## Methods

### Substances

Stock solution of hcrt-1 and hcrt-2 (Tocris, Bristol, UK) were dissolved in pyrogen-free saline (PFS). The non-selective hcrt receptor antagonist, TCS-1102, (Tocris), was dissolved in the vehicle solution prepared by 0.5% saline and 20% D-α-tocopherol polyethylene glycol 1000 succinate (Vitamin E-TPGS; Sigma-Aldrich, St Louis, MO) in PFS. The stock solutions were stored at −20 °C until used. The doses employed in these experiments were as follows: 1 μg and 10 μg for hcrt-1; 1 μg and 10 μg for hcrt-2; 1.25 μg and 2.5 μg for TCS-1102. The total volume of each microinjection was 0.5 μl. Ibuprofen (0.15 mg/ml, Yung-Shin Pharm, Taichung, Taiwan) was dissolved in drinking water and used for controlling post-surgical pain.

### Animal preparations

Male Wistar rats (250–300 g; National Laboratory Animal Breeding and Research Center, Taiwan) were used in present study. These animals were kept in collective plastic cages (3–4 rats/cage) for at least seven days before stereotaxic surgery with the food and water *ad libitum*. Rats were maintained in a sound-attenuated, temperature-controlled (23 ± 1 °C) and light/dark cycle-regulated room (12: 12 hours light:dark cycle; 40 Watt × 4 tubes illumination). All experiments and animal care were performed following the principles outlined in the Institutional Animal Care and Use Committee (IACUC) of National Taiwan University; the approved protocol title is: The role of hypocretin in IS footshock stimuli-induced REM sleep disturbance, and the IACUC approval number is: NTU104-EL-00101.

The animals were anesthetized with Zoletil (tiletamine:zolazepam = 1:1; 50 mg/kg, intraperitoneal injection; Virbac, Carros, France). Sleep-wake activity was recorded by polysomnography, and the detailed surgical implantation of recording electrodes are referenced from our previous publication in Journal of Visualized Experiments^48^. An additional microinjection guide cannula (26 gauge, O.D.: 0.46 mm, I.D.: 0.24 mm; Plastics One, Roanoke, VA, USA) was slowly implanted into the bilateral MRNs (the coordinates: AP, −7.9 mm; ML, 0 mm; DV 7.5 mm relative to bregma). The injected substances can reach bilateral MRNs, since the cannula was implanted at the midline as shown in Figs 1B, 6A. These coordinates were adopted from the Paxinos and Watson rat atlas^49^.

On the second postsurgical day, rats were connected to the recording apparatus via a flexible tether (363–363 cable, Plastics One) and were allowed to accommodate to the tether. Experimental protocols, including the sleep recording, IFS and microinjection, were executed after 7 days when rats recovered from surgery. The location of microinjection cannula was confirmed by injecting 0.5% trypan blue dye at the end of experiment. The recording data were included for the subsequent analyses only when the injection target had been confirmed inside the MRNs. Animals were habituated by daily handling and injections of PFS timed to coincide with scheduled experimental administrations.

### Analysis of sleep-wake activity

Postacquisition determination of the vigilance state was done by visual scoring of 12-s epochs using custom software (ICELUS, M. R. Opp) written in LabView for Windows (National Instruments). The animal’s behavior was classified as either NREM sleep, REM sleep or waking based on previously defined criteria^10^. Briefly, NREM sleep is characterized by large-amplitude EEG slow waves, high power density values in the delta frequency band (0.5–4.0 Hz), reduced muscle tone, and lack of locomotion activity. During REM sleep, the amplitude of the EEG is reduced, the predominant EEG power density occurs within the theta frequency (6.0–9.0 Hz), lowest muscle tone, and there is no locomotion but with some phasic body twitches. During wakefulness, there are protracted locomotion activities and highest muscle tone. The amplitude of EEG is similar to that observed during REMS, but power density values in the delta frequency band are generally greater than those in theta frequency band.

### Manipulation of IFS protocol

A custom-made electrical foot stimulation box (40 cm × 22 cm × 29 cm) was used to initiate an acute stress. The manipulation of IFS protocol lasted for 10 minutes and consisted of twelve times of electrical stimuli, which were randomly given within this 10-min period. The intensity of current for each footshock stimulus was 5.0 mA and the stimulation duration was 50 ms.

### Experimental protocol

Total of 69 Wistar rats were divided into 5 groups. Rats in all groups received 0.5 μl of PFS 15 minutes prior to the beginning of the light period and the sleep-wake activities were collected for 24 hours on the 1^st^ experimental day. On the 2^nd^ experimental day, rats in group 1 (n = 7) were placed in the footshock box for 10 minutes and received the IFS after the PFS injection. Rats in group 2 (n = 7) received 20% TPGS on the 2^nd^ experimental day. The IFS was performed twice on the 3^rd^- and 12^th^-days, and TCS-1102 (1.25 or 2.5 μg) was administered into the MRNs before IFS. The doses of TCS-1102 were randomly assigned to rats on the 3^rd^- and 12^th^-days, and all rats were treated with two different doses. In order to determine the effect of TCS-1102 on the physiological sleep-wake activity, rats received two different doses (1.25 μg and 2.5 μg) of TCS-1102 in two distinct days, the 6^th^- and 9^th^- days. The dosage of each injection day for each rat was selected randomly. Rats in the group 3 (n = 7) received both TPGS injection and IFS on the 2^nd^-day, and two different doses (1 or 10 μg) of hcrt-1 and hcrt-2 were randomly administered in the following four different days. Sleep-wake activities of rats were recorded in their home cages. Rats in group 4 (n = 12) were randomly divided into 2 subgroups; one subgroup was used to determine the expression of prepro-hcrt and hcrt-1, with or without the IFS, at three different zeitgeber times (ZT), ZT0, ZT1 and ZT2; the other subgroup was used to determine the expression of c-fos and hcrt-1, with or without the IFS, at ZT0, ZT1 and ZT2. The manipulation of IFS was performed 10 minutes prior to ZT0. Rats in group 5 (n = 36) were randomly divided into two groups: one group received the IFS and another group did not receive IFS. Rats were sacrificed at ZT0 (n = 6 for each group), ZT1 (n = 6 for each group) and ZT2 (n = 6 for each group), and the concentrations of hypocretin in the hypothalamus were determined by ELISA. The diagram of experimental protocol is depicted in Fig. 6C.

### Immunofluorescence assay (IFA) for prepro-hypocretin, hypocretin-1 and c-fos

The brain tissues were fixed with 4% of paraformaldehyde for 4 hours, and dehydration for 24 hours in 30% sucrose. A serial of brain slices containing LHA from the rostral to caudal sections of LHA were sliced at 30 μm thickness by frozen section. The tissue was first rinsed in phosphate buffered saline (PBS) for 15 minutes, then incubated in PBS with 0.3% of Trition X-100 (PBST) for 30 minutes at room temperature. To reduce the nonspecific background, the slices were stained by blocking solution, which contained 2% of Bovine Serum Albumin (BSA; Sigma-Aldrich, St. Louis, MO, USA) and 5% of Normal goat serum (NGS; Jackson ImmunoResearch, PA, USA) in PBST, and blocking for 2 hours at room temperature. The primary and secondary antibodies were diluted in blocking solution. Rabbit anti-prepro-orexin polyclonal antibody (1/75; EMD Millipore Corporation, Temecula, CA, USA), mouse orexin-A monoclonal antibody (1/250; Santa Cruz Biotechnology, Inc., Dallas, TX, USA), and rabbit anti-c-fos polyclonal antibody (1/75; Sigma) were used as the primary antibodies, and staining for 16 hours at 4 °C. The secondary antibodies were incubated for 2 hours at room temperature followed by rinsing in PBST for 1 hour. Goat anti-rabbit IgG (H + L) cross-adsorbed second antibody (1/350; conjugated with Cyanine5, the red fluorescent dye, ThermoFisher Scientific, Rockford, IL, USA) was used for rabbit anti-prepro-orexin polyclonal antibody and rabbit anti-c-fos polyclonal antibody; antibody-CF^TM^488A Conjugates (1/600; with green fluorescent dye, Sigma) was used for mouse orexin-A monoclonal antibody. 4’,6-Diamidino-2-phenylindole (DAPI), a blue fluorescent nuclei acid stain, was used to stain cell nuclei (Sigma). The subsequent procedures consisted of rinsing in PBS for 15 minutes, then mounted slides with DPX Mountant for histology (Sigma). The sections were then examined under microscope (Olympus IX83, Tokyo, Japan) and the images were photographed by DP80 (Olympus). The positive staining cells were counted from all of the sections by ImageJ (National Institutes of Health, MD, USA).

### ELISA for hypocretin

Rat hypocretin ELISA kits were obtained from Wuhan Fine Biotech Co., Ltd. (Wuhan, China) and the procedures followed the standard instructions provided by the manufacturers. Absorbance was measured by an ELISA plate reader (Multiskan EX, Thermo Electron Corp., Waltham, MA, USA) with the wavelength set at 450 nm. The sensitivity is <4.688 pg mL^−1^, the assay range is between 7.8 and 500 pg mL^−1^, the intra-assay coefficient of variation is <8% and the inter-assay coefficient of variation is <10%.

### Statistical and data analyses

All values acquired from sleep-wake recordings were presented as the mean ± standard error of the mean (SEM) for the indicated sample sizes. Two-way repeated measures analyses of variance (ANOVA) for the duration of each vigilance state (NREM sleep, REM sleep, and wakefulness), for sleep architecture parameters, and for slow wave activities (SWAs) were performed by comparing before and after manipulation within the group and between groups, and across the two 12-hour time blocks as mentioned later in the result section. We used Scheffe *post hoc* comparison to process multiple comparisons among treated groups for vigilance states, sleep architecture parameters and slow wave activities. Unpaired student t-test was performed to analyze ELISA results. An α level of p < 0.05 was taken as indicating a statistically significant difference. If statistically significant differences were detected, a Scheffe *post hoc* comparison was made to determine which values during experimental conditions deviated from those obtained from the control conditions.

## References

[1] Lupien SJ, McEwen BS, Gunnar MR, Heim C (2009). Effects of stress throughout the lifespan on the brain, behaviour and cognition. Nat. Rev. Neurosci..

[2] Ulrich-Lai YM, Herman JP (2009). Neural regulation of endocrine and autonomic stress responses. Nat. Rev. Neurosci..

[3] Baglioni C, Spiegelhalder K, Lombardo C, Riemann D (2010). Sleep and emotions: a focus on insomnia. Sleep Med. Rev..

[4] Cui R, Li B, Suemaru K, Araki H (2007). Differential effects of psychological and physical stress on the sleep pattern in rats. Acta Med. Okayama..

[5] Maclean RR, Datta S (2007). The relationship between anxiety and sleep-wake behavior after stressor exposure in the rat. Brain Res..

[6] Pawlyk AC, Morrison AR, Ross RJ, Brennan FX (2008). Stress-induced changes in sleep in rodents: models and mechanisms. Neurosci. Biobehav. Rev..

[7] Sanford LD, Yang L, Wellman LL, Liu X, Tang X (2010). Differential effects of controllable and uncontrollable footshock stress on sleep in mice. Sleep.

[8] Yang L, Wellman LL, Tang X, Sanford LD (2011). Effects of corticotropin releasing factor (CRF) on sleep and body temperature following controllable footshock stress in mice. Physiol. Behav..

[9] Hsiao YT, Yi PL, Li CL, Chang FC (2011). Effect of cannabidiol on sleep disruption induced by the repeated combination tests consisting of open field and elevated plus-maze in rats. Neuropharmacology.

[10] Chang FC, Opp MR (1998). Blockade of corticotropin-releasing hormone receptors reduces spontaneous waking in the rat. Am. J. Physiol. Regul. Integr. Comp. Physiol..

[11] Berridge CW, España RA, Vittoz NM (2010). Hypocretin/orexin in arousal and stress. Brain Res..

[12] Hsiao YT, Jou SB, Yi PL, Chang FC (2012). Activation of GABAergic pathway by hypocretin in the median raphe nucleus (MRN) mediates stress-induced theta rhythm in rats. Behav. Brain Res..

[13] Bourgin P (2000). Hypocretin-1 modulates rapid eye movement sleep through activation of locus coeruleus neurons. J. Neurosci..

[14] Xi MC, Morales FR, Chase MH (2001). Effects on sleep and wakefulness of the injection of hypocretin-1 (orexin-A) into the laterodorsal tegmental nucleus of the cat. Brain Res..

[15] Chemelli RM (1999). Narcolepsy in orexin knockout mice: molecular genetics of sleep regulation. Cell.

[16] Nishino S, Ripley B, Overeem S, Lammers GJ, Mignot E (2000). Hypocretin (orexin) deficiency in human narcolepsy. Lancet.

[17] Nambu T (1999). Distribution of orexin neurons in the adult rat brain. Brain Res..

[18] Marcus JN (2001). Differential expression of orexin receptors 1 and 2 in the rat brain. J. Comp. Neurol..

[19] Greco MA, Shiromani PJ (2001). Hypocretin receptor protein and mRNA expression in the dorsolateral pons of rats. Brain Res. Mol. Brain Res..

[20] Marston OJ (2008). Circadian and dark-pulse activation of orexin/hypocretin neurons. Mol. Brain..

[21] Grove G, Coplan JD, Hollander E (1997). The neuroanatomy of 5-HT dysregulation and panic disorder. J. Neuropsychiatry Clin. Neurosci..

[22] Srebro B, Lorens SA (1975). Behavioral effects of selective midbrain raphe lesions in the rat. Brain Res..

[23] Graeff FG, Silveira Filho NG (1978). Behavioral inhibition induced by electrical stimulation of the median raphe nucleus of the rat. Physiol. Behav..

[24] Avanzi V, Castilho VM, de Andrade TG, Brandao ML (1998). Regulation of contextual conditioning by the median raphe nucleus. Brain Res..

[25] Andrade TG, Macedo CE, Zangrossi H, Graeff FG (2004). Anxiolytic-like effects of median raphe nucleus lesion in the elevated T-maze. Behav. Brain Res..

[26] Jacobs BL, Fornal CA (1991). Activity of brain serotonergic neurons in the behaving animal. Pharmacol. Rev..

[27] Arpa J, De Andres I (1993). Re-examination of the effects of raphe lesions on the sleep/wakefulness cycle states in cats. J. Sleep Res..

[28] Meyer-Bernstein EL, Morin LP (1999). Electrical stimulation of the median or dorsal raphe nuclei reduces light-induced FOS protein in the suprachiasmatic nucleus and causes circadian activity rhythm phase shifts. Neuroscience.

[29] Muscat L, Tischler RC, Morin LP (2005). Functional analysis of the role of the median raphe as a regulator of hamster circadian system sensitivity to light. Brain Res..

[30] Sheu YS, Nelson JP, Bloom FE (1974). Discharge patterns of cat raphe neurons during sleep and waking. Brain Res..

[31] Maloney KJ, Mainville L, Jones BE (1999). Differential c-Fos expression in cholinergic, monoaminergic, and GABAergic cell groups of the pontomesencephalic tegmentum after paradoxical sleep deprivation and recovery. J. Neurosci..

[32] Chang FC, Opp MR (2002). Role of corticotropin-releasing hormone in stressor-induced alterations of sleep in rat. Am. J. Physiol. Regul. Integr. Comp. Physiol..

[33] Tang X, Xiao J, Liu X, Sanford LD (2004). Strain differences in the influence of open field exposure on sleep in mice. Behav. Brain Res..

[34] Pawlyk AC, Jha SK, Brennan FX, Morrison AR, Ross RJ (2005). A rodent model of sleep disturbances in posttraumatic stress disorder: the role of context after fear conditioning. Biol. Psychiatry..

[35] Lauer CJ, Krieg JC, Garcia-Borreguero D, Ozdaglar A, Holsboer F (1992). Panic disorder and major depression: a comparative electroencephalographic sleep study. Psychiatry Res..

[36] Habukawa M, Uchimura N, Maeda M, Kotorii N, Maeda H (2007). Sleep findings in young adult patients with posttraumatic stress disorder. Biol. Psychiatry..

[37] Lavie P, Hefez A, Halperin G, Enoch D (1979). Long-term effects of traumatic war-related events on sleep. Am. J. Psychiatry..

[38] Hefez A, Metz L, Lavie P (1987). Long-term effects of extreme situational stress on sleep and dreaming. Am. J. Psychiatry..

[39] Ross RJ (1994). Rapid eye movement sleep disturbance in posttraumatic stress disorder. Biol. Psychiatry.

[40] Breslau N (2004). Sleep in lifetime posttraumatic stress disorder: a community-based polysomnographic study. Arch Gen. Psychiatry.

[41] Bonnet C, Leger L, Baubet V, Debilly G, Cespuglio R (1997). Influence of a 1 h immobilization stress on sleep states and corticotropin-like intermediate lobe peptide (CLIP or ACTH18-39, Ph-ACTH18-39) brain contents in the rat. Brain Res..

[42] Dewasmes G, Loos N, Delanaud S, Dewasmes D, Ramadan W (2004). Pattern of rapid-eye movement sleep episode occurrence after an immobilization stress in the rat. Neurosci. Lett..

[43] Yoshida Y (2001). Fluctuation of extracellular hypocretin-1 (orexin A) levels in the rat in relation to the light-dark cycle and sleep-wake activities. Eur. J. Neurosci..

[44] Ida T (2000). Possible involvement of orexin in the stress reaction in rats. Biochem. Biophys. Res. Commun..

[45] Sakamoto F, Yamada S, Ueta Y (2004). Centrally administered orexin-A activates corticotropin-releasing factor-containing neurons in the hypothalamic paraventricular nucleus and central amygdaloid nucleus of rats: possible involvement of central orexins on stress-activated central CRF neurons. Regul. Pept..

[46] Mieda M (2011). Differential roles of orexin receptor-1 and -2 in the regulation of non-REM and REM sleep. J. Neurosci..

[47] Sutcliffe JG, de Lecea L (2002). The hypocretins: setting the arousal threshold. Nat. Rev. Neurosci. 2002.

[48] Yi, P. L., Jou, S. B., Wu, Y. J. & Chang, F. C. Manipulation of epileptiform ECoGs and sleep in rats and mice by acupuncture. *J. Vis. Exp*. **118**, 10.3791/54896 (2016). 10.3791/54896 PMC5226448 28060294

[49] Paxinos, G. & Watson, C. The rat brain in stereotaxic coordinates. San Diego: Academic Press (1998).

